# Aluminum Exposure from Parenteral Nutrition: Early Bile Canaliculus Changes of the Hepatocyte

**DOI:** 10.3390/nu10060723

**Published:** 2018-06-04

**Authors:** Amanda R. Hall, Ha Le, Chris Arnold, Janet Brunton, Robert Bertolo, Grant G. Miller, Gordon A. Zello, Consolato Sergi

**Affiliations:** 1 Department of Surgery, University of Saskatchewan, Saskatoon, SK S7N 5A2, Canada; arb743@mail.usask.ca (A.R.H.); grant.miller@usask.ca (G.G.M.); 2 Department of Community Health & Epidemiology, University of Saskatchewan, Saskatoon, SK S7N 5A2, Canada; htl842@mail.usask.ca; 3 College of Pharmacy and Nutrition, University of Saskatchewan, Saskatoon, SK S7N 5A2, Canada; chris.arnold@saskatoonhealthregion.ca (C.A.); gaz511@campus.usask.ca (G.A.Z.); 4 Department of Biochemistry, Memorial University of Newfoundland, St. John’s, NL A1B 3X9, Canada; jbrunton@mun.ca (J.B.); rbertolo@mun.ca (R.B.); 5 Department of Lab. Medicine and Pathology, University of Alberta, Edmonton, AB T6G 2B7, Canada; 6 Department of Pediatrics, Stollery Children’s Hospital, Edmonton, AB T6G 2B7, Canada

**Keywords:** aluminum, exposure, parenteral, toxicity, liver, canaliculus

## Abstract

*Background:* Neonates on long-term parenteral nutrition (PN) may develop parenteral nutrition-associated liver disease (PNALD). Aluminum (Al) is a known contaminant of infant PN, and we hypothesize that it substantially contributes to PNALD. In this study, we aim to assess the impact of Al on hepatocytes in a piglet model. *Methods:* We conducted a randomized control trial using a Yucatan piglet PN model. Piglets, aged 3–6 days, were placed into two groups. The high Al group (*n* = 8) received PN with 63 µg/kg/day of Al, while the low Al group (*n* = 7) received PN with 24 µg/kg/day of Al. Serum samples for total bile acids (TBA) were collected over two weeks, and liver tissue was obtained at the end of the experiment. Bile canaliculus morphometry were studied by transmission electron microscopy (TEM) and ImageJ software analysis. *Results:* The canalicular space was smaller and the microvilli were shorter in the high Al group than in the low Al group. There was no difference in the TBA between the groups. *Conclusions:* Al causes structural changes in the hepatocytes despite unaltered serum bile acids. High Al in PN is associated with short microvilli, which could decrease the functional excretion area of the hepatocytes and impair bile flow.

## 1. Introduction

The typical premature infant receiving parenteral nutrition (PN) is exposed to Al in amounts that far exceed safe limits [1,2]. This exposure is further aggravated by the reduced Al excretion of their underdeveloped kidneys [3,4]. In addition to the brain and bone [5], Al is also deposited in the liver, where it could be toxic to the hepatocyte. At an ultrastructural level, Al is seen in hepatic lysosomes and other organelles, and is speculated to cause structural changes that may impair biliary secretion [6,7,8,9]. Al forms a superoxide radical that disrupts mitochondrial function and triggers apoptosis [10,11], but the hepatotoxic mechanism of this element is poorly understood.

Most of the previous work looking at the hepatoxicity of Al in PN has been done with Intralipid^®^, a soy-based lipid emulsion with a ω-6 to ω-3 ratio of 7:1. Omega-6 lipids are relatively pro-inflammatory and are speculated to be a major contributing factor to parenteral nutrition-associated liver disease (PNALD). [12,13,14]. Smoflipid^®^ (Fresenius Kabi, Homburg, Germany) has reduced ω-6 fatty acids with an ω-6:ω-3 of 2.5:1 and seven times less phytosterols than Intralipid^®^. Both the change in lipids and phytosterols may deter the development of PNALD [15]. Our goal was to evaluate the hepatotoxicity of Al in subjects receiving PN with Smoflipid^®^. We hypothesized that parenteral Al would be hepatoxic independent of the type of lipid in PN. This pilot study uses a Yucatan miniature piglet Smoflipid^®^-based PN model to observe the effects of two different levels of Al contamination on the ultrastructure of hepatocytes.

## 2. Materials and Methods

### 2.1. Animal Work

This study was approved by both the University of Saskatchewan Animal Research Ethics Board and the Institutional Animal Care Committee at Memorial University of Newfoundland. Yucatan miniature piglets, aged 3–6 days, were placed randomly into one of two groups. A high Al (HiAl) group (*n* = 8) received PN with 63 μg/kg/day of Al, while the low Al (LoAl) group (*n* = 7) received otherwise identical PN with 24 μg/kg/day of Al. The amount of Al in the latter category is within the range of contamination found in Canadian neonatal PN [16]. A group of four piglets (Reference group) was also maintained on a standard oral diet and used as a reference for hepatic ultrastructure, but this group was not included in the main analysis because of the small size of the Reference group.

On day zero, the piglets underwent general anesthesia, and central venous catheters were implanted, tunneled out the piglet’s back, and secured in a tether and swivel system. PN was started on the day of surgery and increased incrementally over 24 h to a goal rate of 12 mL/kg/h. Prophylactic antibiotics were given every second day. The piglets were housed in individual metabolic cages and exposed to standard 12 h light/dark cycles.

The piglets were kept on a strict PN regimen for the 14 days of the study. Both groups received identical PN formulation, including Smoflipid^®^. The Smoflipid^®^ was administered at 1.9 mL/kg/h (45.6 mL/kg/day). The amino acid-dextrose solution was infused at a rate of 10.1 mL/kg/h (242.4 mL/kg/day). Every second day the piglets were weighed, and the PN rate was adjusted to maintain adequate nutrient delivery. Every fourth day, serum samples were collected. At the end of the 14 days, the piglets were euthanized and liver samples were collected. The dose of Smoflipid^®^ administered to piglet is 3–5 times the usual doses administered to human neonates (9.1 vs. 2–3 g/kg/day). This would increase the omega-3 intake significantly compared to that used clinically, which may affect the development of hepatic injury. The piglet grows at ~5 times the rate of infants, so the requirements for all nutrients are extrapolated by growth rate. In PN studies of amino acid requirements, we predict the infant requirement by dividing the piglet requirement by 5 as explained in detail in Chapman et al. [17]. Thus, it makes sense the Smoflipid^®^ dose is 3–5 times higher to accommodate the 5-times-higher energy needs.

### 2.2. Transmission Electron Microscopy 

**Figure 1:**
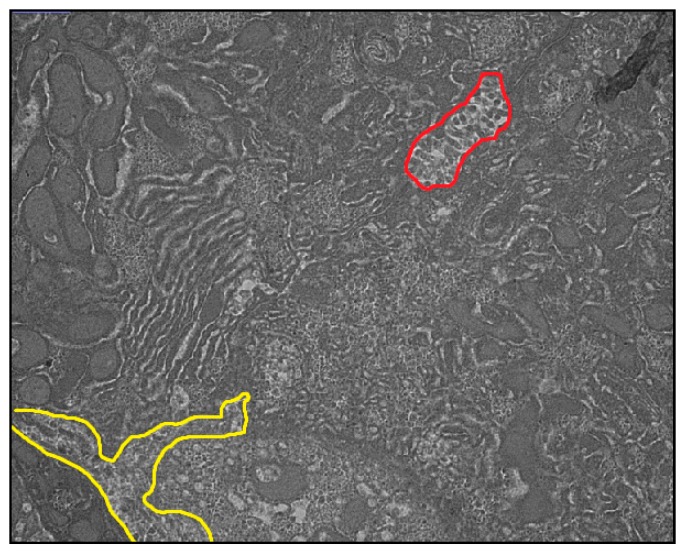
Illustrates an example of mapping using ultrathin sections of transmission electron microscopy (TEM). Red line surrounds the canalicular space, and the yellow lines are around the area of Disse. The ImageJ software uses these boundaries to calculate morphometric parameters automatically.

Liver samples were fixed, dehydrated, and resin-embedded using a standard sodium cacodylate and osmium tetroxide-based technique [18]. Uranyl acetate and lead citrate-stained ultra-thin sections (70–90 nm) were viewed and photographed under an electron microscope with energy-dispersive spectrometer capability (Hitachi HT7700, Tokyo, Japan), operating at an accelerating voltage of 80 kV. For each piglet, the three images of the clearest bile canaliculi and space of Disse were analyzed using the open access ImageJ software program (U.S. National Institutes of Health, Bethesda, MD, USA). For a selected image, ImageJ can calculate the length and area in pixel values and convert to microns (Figure 1) [19]. To minimize bias, we sampled multiple different areas of each liver and took our measurements from three different microphotographs for each piglet.

### 2.3. Bile Acid Assay

We used a colorimetric kit (BQKits, San Diego, CA, USA) for the total bile acid assay, following the manufacturer’s instructions. Each serum sample was tested twice, and any sample with variance higher than 10% was re-tested.

### 2.4. Statistical Analysis

All statistical analysis was carried out using SPSS version 22 (IBM, Armonk, NY, USA) and validated by SAS software (SAS Institute, Cary, NC, USA). The bile acid assay results were examined using a repeated measurement model for longitudinal analysis, paired with non-parametric tests for the study of data on separate days (i.e., all pigs on day 4). The measurements obtained from the transmission electron microscopy were compared between the groups using T-tests and Chi-square analysis. A *p*-value of less than 0.05 was considered statistically significant for all tests.

## 3. Results

**Figure 2:**
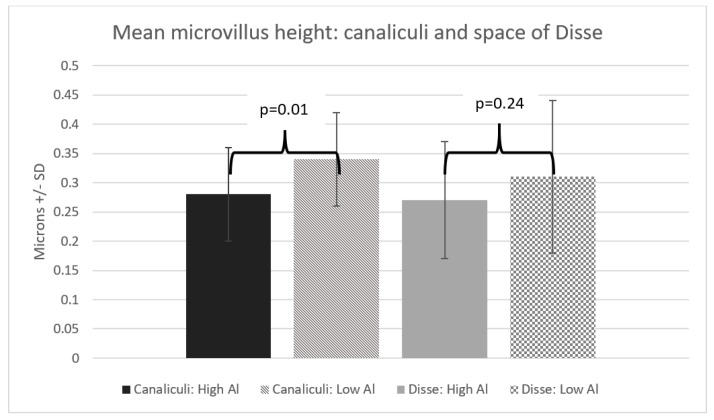
Mean microvillus height: canaliculi and space of Disse.

**Figure 3:**
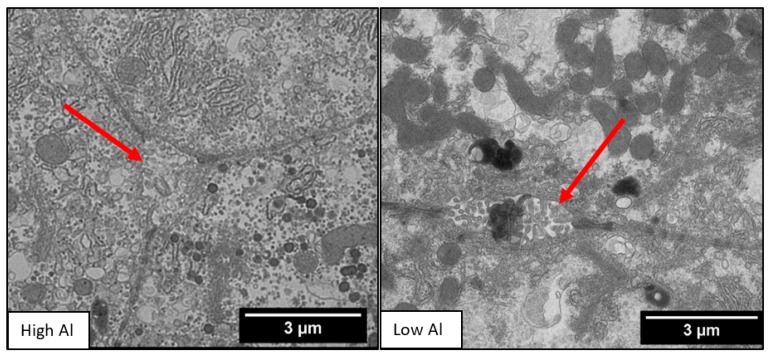
Low vs. High Al transmission electron micrographs with the arrows pointing specifically to the canalicular space. Note that the microvilli in the Low Al canaliculi are longer than in the High Al canaliculi. Both transmission electron micrographs have been taken at the same magnification.

**Figure 4:**
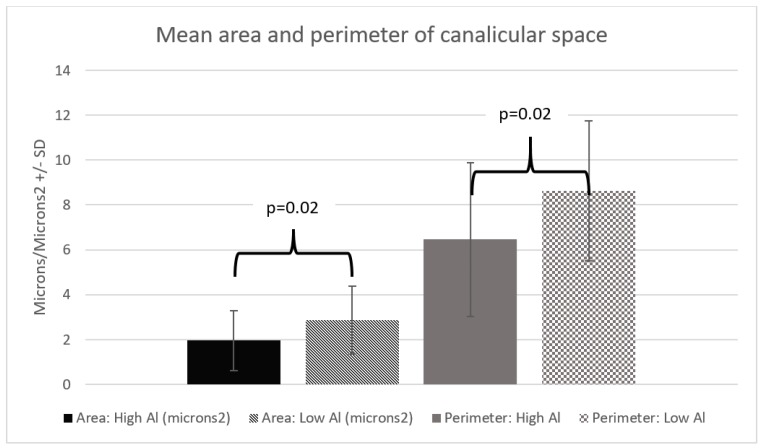
Mean area and perimeter of canalicular space.

**Figure 5:**
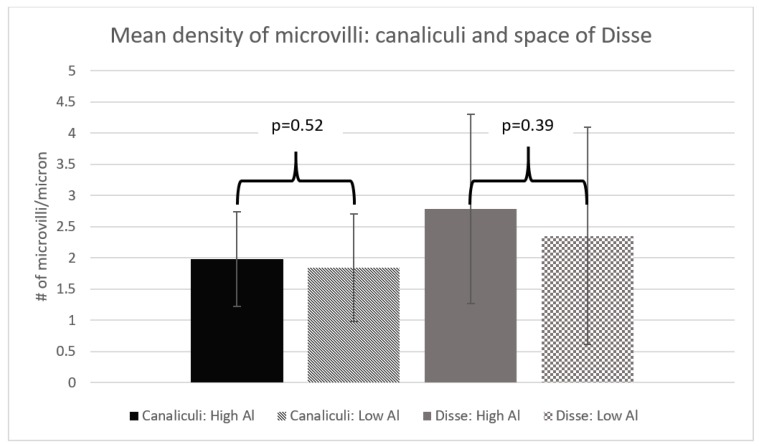
Mean density of microvilli: Canalicular and space of Disse.

Our two groups had similar characteristics (Table 1). We found the microvilli significantly shorter, with a mean difference of 0.06 µm (*p* = 0.01) in the HiAl group as compared to the LoAl group. Microvilli density was similar between the two groups. The area and the perimeter length of the canalicular space was significantly smaller in the HiAl as compared to the LoAl group, with mean differences of 0.92 µm^2^ and 2.17 µm respectively (*p* = 0.02 for both canalicular perimeter and area). There was no significant difference in the width of the space of Disse (*p* = 0.44), and likewise, no differences in the Disse microvilli density or length (Figure 2, Figure 3, Figure 4 and Figure 5).

**Figure 6:**
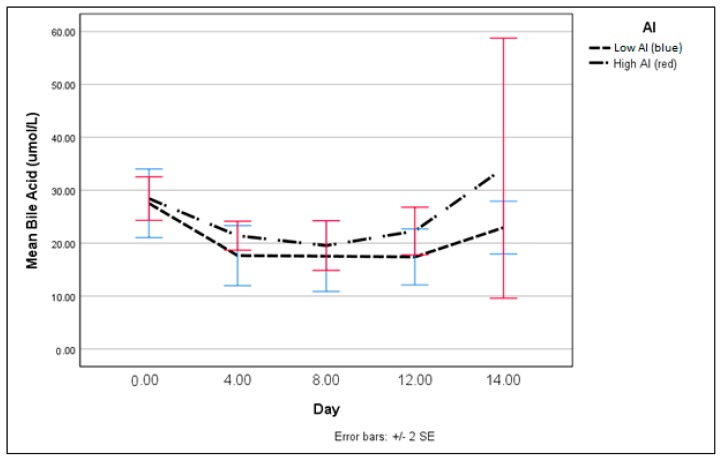
Total serum bile acids: High vs. Low Al.

Our repeated measurement model for the serum bile acids showed no significant difference in the slopes (*p* = 0.48) or between the values for each day (Figure 6).

Moreover, we noted a higher number of electron-dense lesions in the mitochondria for only the HiAl group, when compared to healthy piglets (Table 2).

## 4. Discussion

In our study, Al was associated with blunting of the canalicular microvilli and this appeared to be dose-dependent. Despite the use of a mixed lipid in the PN solutions, we observed significantly shorter canalicular microvilli in the HiAl group as compared to the LoAl group. Canalicular microvilli are essential components of bile excretion and shortening or loss of these structures is one of the first histologic changes of cholestasis [20,21,22]. The apical bile acid transporters, such as Mrp2 and Bsep, are located on the microvilli [23]. If the canalicular microvilli are blunted, then there is less surface area for bile acid transporters and their ability to excrete bile could be impaired. Al in PN caused shortened microvilli in previous piglet PN models, but these studies used only soy-based lipids in PN [24]. Our study is the first demonstrating that Al may be an independent factor in microvilli blunting, although further research is required to compare the two types of lipids and Al.

Al is known to cause oxidative stress in a variety of organ systems [25,26,27] and we can speculate that this is the likely reason for the negative effects of Al in our model. We noted a higher number of electron-dense lesions in the mitochondria for only the HiAl group, when compared to healthy piglets. Although the Reference group is small, and the statistical difference should be interpreted cautiously, it is still a finding deserving of further research. The difference we observed is consistent with other studies showing that mitochondria accumulate electron-dense deposits when the cell is facing oxidative stress, such as with ischemic/reperfusion injury [28,29,30]. These mitochondrial deposits also appear in other chronic liver conditions, such as Wilson’s disease [31,32].

Overall, there is a paucity of work using TEM to study PNALD, but at least one PNALD study has found mitochondrial changes and loss of microvilli, in conjunction with rising markers of oxidative stress, similar to our animal model [33,34,35]. Oxidative stress is responsible for the production of proinflammatory cytokines, produced by lymphocytes and Kupffer cells, among which tumor necrosis factor alpha, transforming growth factors alpha and beta, interleukins 6 and 8, nuclear factor-kappa B, and adiponectin are prominent. In mitochondrial cardiomyopathies, these cytokines are produced through free radical-mediated mechanisms, by altering mitochondrial membrane permeability and inhibiting the respiratory chain with a mechanism that seems to be ubiquitous for several cell types [36,37,38,39,40,41,42].

The canalicular spaces were also larger in the LoAl group. Previously, we found that a higher Al exposure caused a ballooning of canalicular space [24]. Ballooning of the canalicular space is seen in animal models of different cholestatic scenarios, including PNALD [20,43], and inflammatory cholestasis [43]. The Smoflipid^®^ given to both of our groups was not used in any of these previous PN studies and perhaps this less-inflammatory lipid prevented ballooning of the canaliculus [24,44]. The impact of smaller canalicular spaces is still under investigation.

Neither group developed elevated serum bile acids, nor was there a difference in the total serum bile acids between the two Al groups. This was unexpected, as other piglet PN studies have shown high serum bile acids within two weeks of PN exposure, but these studies used Intralipid^®^ in PN [45]. Our use of Smoflipid^®^ may have delayed the onset of a rise in serum bile acids, as this lipid is known to delay the rise of clinical PNALD markers, such as liver enzymes and serum bile acids [46,47]. The lack of rise in total serum bile acids is important because these types of serum biochemical changes are used as one of the earliest indicators of PNALD in a clinical setting. In this investigation, significant ultrastructural changes occurred in hepatocytes without (and likely before) the accumulation of high serum total bile acids, indicating that Al may play a role in the most preliminary stages of PNALD.

A limitation of this study is the small number of exposed piglets and reference group. However, we think that our results support the case that further research into the long-term effects of Al toxicity is needed, as are techniques to decrease the contamination of Al in infant PN. The use of more detailed definition using ultrastructural analysis has been a pillar of previous studies on ischemia-reperfusion injury of a liver transplantation model and in an intestinal failure-associated liver disease [21,22,44,48]. In the future, we may target the transporters of the bile canaliculus using gold-labeled immune antibodies for electron microscopy.

The piglet is the most suitable choice for a PNALD animal model because it closely mimics infant physiology, anatomy, and metabolism. PNALD is a disease of prematurity, and both the human infant and the piglet are born with underdeveloped gastrointestinal/hepatic systems. Although piglets require significantly more calories because of their expedited growth, the nutritional needs between the two species are otherwise very similar and piglets have been used for decades to test newborn human formulas [49,50,51].

The majority of Al in PN comes from calcium gluconate and other components, which are stored in glass containers. This type of glass packaging leaks Al into the solution in a time-dependent fashion [52,53,54]. There are many potential methods to reduce Al in PN formulations. Most commonly, PN products list the amount of Al expected at expiry of the product, but multiple studies have demonstrated that this measurement is highly inaccurate and of little use to compounding pharmacists [1]. Additionally, compounding issues such as acidosis and phosphate precipitation prohibit the substitution of calcium chloride for calcium gluconate [55,56]. The most effective method is to eliminate the glass packaging completely. Some regions, such as the UK and Canada, have mandated that calcium gluconate injectable solutions for use in pediatric patients be stored in polyethylene containers instead of glass vials [57].

## 5. Conclusions

Al causes shortening of canalicular microvilli, without and potentially before the elevation of serum total bile acids. This implies that a rise in serum bile acids may not be the first indicator of hepatic damage. Additionally, the use of a less inflammatory lipid in PN did not prevent Al-induced ultrastructural changes. Plausibly, with more prolonged exposure, the lower Al-containing PN could also cause deleterious changes to the hepatocytes.

## Figures and Tables

**Table 1:** Characteristics of the study population and ANOVA analysis.

	High Al (*N* = 8)	Standard Al (*N* = 7)	Reference (*N* = 4)	Difference (*p* Value)
Mean starting age (days + SD)	8.38 (±2.92)	7.71 (±2.43)	10.25 (±1.50)	*p* = 0.20
Mean starting weight (kg+ SD)	1.58 (±0.14)	1.57 (±0.15)	1.32 (±0.24)	*p* = 0.06
Females: Males	5:3	3:4	3:1	*p* = 0.60

**Table 2:** TEM measurements and ANOVA analysis (see Supplementary Materials Table S1 for full data on TEM measurements).

	High Al	Standard Al	Reference	Difference (*p* Value)
Mean number of high density lesions in mitochondria per hepatocyte (±SD)	1.58 (±0.88)	1.43 (±0.68)	0.42 (±0.52)	*p* < 0.001

## Supplementary Materials

The following are available online at http://www.mdpi.com/2072-6643/10/6/723/s1, Table S1: TEM Measurements and ANOVA analysis.
