# Supplementary material for: Aluminum Exposure from Parenteral Nutrition: Early Bile Canaliculus Changes of the Hepatocyte
Source: Nutrients. 2018 Jun 4;10(6):723. doi: 10.3390/nu10060723 (PMC6024673; doi:10.3390/nu10060723)
Supplement: Supplementary file 1 [file nutrients-10-00723-s001.pdf]

Table S1: Full TEM Measurements and ANOVA analysis.

	High Al	Standard Al	Reference	Difference (p value)
**Canalicular Space**				
Mean canalicular area (+/- SD) (microns^2)	1.96 (+/- 1.33)	2.88 (+/- 1.51)	1.47 (+/-0.66)	p= 0.001
Mean canalicular perimeter (+/- SD) (microns)	6.46 (+/- 3.44)	8.64 (+/- 3.12)	6.39 (+/- 2.51)	p=0.021
Mean microvillus height (+/- SD) (microns)	0.28 (+/- 0.08)	0.34 (+/- 0.08)	0.33 (+/-0.07)	p=0.021
Mean density of microvilli per canaliculi (+/- SD) (# of microvilli/ micron)	3.90 (+/- 0.84)	4.16 (+/- 0.94)	4.39 (+/-0.78)	p=0.16
Mean number of microvilli per canaliculus (+/- SD)	12.40 (+/- 7.52)	16.00 (+/- 10.79)	11.94 (+/- 6.06)	p=0.20
**Space of Disse**				
Mean width of space of Disse (+/- SD) (microns)	0.68 (+/-0.32)	0.76 (+/- 0.34)	0.60 (+/- 0.19)	p=0.35
Mean canalicular height (+/-SD) (microns)	0.27 (+/-0.10)	0.31 (+/- 0.13)	0.38 (+/- 0.10)	p=0.024
Mean density of microvilli in space of Disse (+/- SD) (# of microvilli/ micron)	2.78 (+/- 1.52)	2.35 (+/- 1.74)	3.08 (+/- 0.75)	p=0.38
**Miscellaneous**				
Mean number of high density lesions in mitochondria per hepatocyte (+/- SD)	1.58 (+/- 0.88)	1.43 (+/- 0.68)	0.42 (+/- 0.52)	p<0.001
